# Drawing Dynamical and Parameters Planes of Iterative Families and Methods

**DOI:** 10.1155/2013/780153

**Published:** 2013-11-24

**Authors:** Francisco I. Chicharro, Alicia Cordero, Juan R. Torregrosa

**Affiliations:** ^1^Instituto de Telecomunicaciones y Aplicaciones Multimedia, Universitat Politècnica de València, Camino de Vera s/n, 46022 Valencia, Spain; ^2^Instituto de Matemática Multidisciplinar, Universitat Politècnica de València, Camino de Vera s/n, 46022 Valencia, Spain

## Abstract

The complex dynamical analysis of the parametric fourth-order Kim's iterative family is made on quadratic polynomials, showing the MATLAB codes generated to draw the fractal images necessary to complete the study. The parameter spaces associated with the free critical points have been analyzed, showing the stable (and unstable) regions where the selection of the parameter will provide us the excellent schemes (or dreadful ones).

## 1. Introduction

It is usual to find nonlinear equations in the modelization of many scientific and engineering problems, and a broadly extended tools to solve them are the iterative methods. In the last years, it has become an increasing and fruitful area of research. More recently, complex dynamics has been revealed as a very useful tool to deep in the understanding of the rational functions that rise when an iterative scheme is applied to solve the nonlinear equation *f*(*z*) = 0, with *f* : *ℂ* → *ℂ*. The dynamical properties of this rational function give us important information about numerical features of the method as its stability and reliability.

There is an extensive literature on the study of iteration of rational mappings of complex variables (see [1, 2], for instance). The simplest and more deeply analyzed model is obtained when *f*(*z*) is a quadratic polynomial and the iterative process is Newton's one. The dynamics of this iterative scheme has been widely studied (see, among others, [2–4]).

In the past decade Varona, in [5] and Amat et al. in [6] described the dynamical behavior of several well-known iterative methods. More recently, in [7–14], the authors studied the dynamics of different iterative families. In most of these studies, interesting dynamical planes, including some periodical behavior and other anomalies, have been obtained. In a few cases, the parameter planes have been also analyzed.

In order to study the dynamical behavior of an iterative method when it is applied to a polynomial *p*(*z*), it is necessary to recall some basic dynamical concepts. For a more extensive and comprehensive review of these concepts, see [3, 15].

Let $R:\hat{\mathbb{C}}\to \hat{\mathbb{C}}$ be a rational function, where $\hat{\mathbb{C}}$ is the Riemann sphere. The orbit of a point $z_{0}\in \hat{\mathbb{C}}$ is defined as the set of successive images of *z*_0_ by the rational function, {*z*_0_, *R*(*z*_0_),…, *R* ^*n*^(*z*_0_),…}.

The dynamical behavior of the orbit of a point on the complex plane can be classified depending on its asymptotic behavior. In this way, a point *z*_0_ ∈ *ℂ* is a fixed point of *R* if *R*(*z*_0_) = *z*_0_. A fixed point is attracting, repelling, or neutral if |*R*′(*z*_0_)| is less than, greater than, or equal to 1, respectively. Moreover, if |*R*′(*z*_0_)| = 0, the fixed point is superattracting.

If *z*_f_* is an attracting fixed point of the rational function *R*, its basin of attraction *𝒜*(*z*_f_*) is defined as the set of preimages of any order such that

(1) $$\begin{array}{c} 𝒜\left(z_{f}^{*}\right)=\left\{z_{0}\in \hat{\mathbb{C}}:R^{n}\left(z_{0}\right)\to z_{f}^{*},n\to \infty \right\}. \end{array}$$

The set of points whose orbits tends to an attracting fixed point *z*_f_* is defined as the Fatou set, *ℱ*(*R*). The complementary set, the Julia set *𝒥*(*R*), is the closure of the set consisting of its repelling fixed points and establishes the boundaries between the basins of attraction.

In this paper, Section 2 is devoted to the complex analysis of a known fourth-order family, due to Kim (see [16]). The conjugacy classes of its associated fixed point operator, the stability of the strange fixed points, the analysis of the free critical points, and the analysis of the parameter and dynamical planes are made. In Section 3, the Matlab code used to generate these tools is shown and the key instructions are explained in order to help their eventual modification to adapt them to other iterative families. Finally, some conclusions and the references used in this work are presented.

## 2. Complex Dynamics Features of Kim's Family

We will focus our attention on the dynamical analysis of a known parametric family of fourth-order methods for solving a nonlinear equation *f*(*x*) = 0. Kim in [16] designed a parametric class of optimal eighth-order methods, whose two first steps are

(2) $$\begin{array}{c} y_{k}=x_{k}-\frac{f\left(x_{k}\right)}{f^{\prime }\left(x_{k}\right)},\\ x_{k+1}=y_{k}-\frac{1+\beta u+\lambda u^{2}}{1+\left(\beta -2\right)u+\mu u^{2}}\frac{f\left(y\right)}{f^{\prime }\left(x\right)}, \end{array}$$

where *u* = *f*(*y*)/*f*(*x*). If we suppose *η* = *μ* = 0, the result is a one-parametric family of iterative schemes whose order of convergence is four, for every value of *λ*.

In order to study the affine conjugacy classes of the iterative methods, the following scaling theorem can be easily checked.

**Theorem**

Let *g*(*z*) be an analytic function, and let *A*(*z*) = *αz* + *β*, with *α* ≠ 0, be an affine map. Let *h*(*z*) = *γ*(*g*∘*A*)(*z*), with *γ* ≠ 0. Let *O*_*p*_(*z*) be the fixed point operator of Kim's method on *p*(*z*). Then, *A*∘*O*_*h*_∘*A* ^−1^(*z*) = *O*_*g*_(*z*); that is, *O*_*g*_ and *O*_*h*_ affine conjugated by *A*.

This result allows us to know the behavior of an iterative scheme on a family of polynomials with just the analysis of a few cases, from a suitable scaling.

In the following we will analyze the dynamical behavior of the fourth-order parametric family (2), on quadratic polynomial *p*(*z*) = (*z* − *a*)(*z* − *b*), where *a*, *b* ∈ *ℂ*.

We apply the Möbius transformation

(3) $$\begin{array}{c} M\left(u\right)=\frac{u-a}{u-b}, \end{array}$$

whose inverse is

(4) $$\begin{array}{c} {\left[M\left(u\right)\right]}^{-1}=\frac{ub-a}{u-1}, \end{array}$$

in order to obtain the one-parametric operator

(5) $$\begin{array}{c} O_{p}\left(z,\lambda \right)=-\frac{z^{4}\left(1-\lambda +4z+6z^{2}+4z^{3}+z^{4}\right)}{-1-4z-6z^{2}-4z^{3}+\left(-1+\lambda \right)z^{4}}, \end{array}$$

associated with the iterative method. In the study of the rational function (5), *z* = 0 and *z* = *∞* appear as superattracting fixed points and *z* = 1 is a strange fixed point for *λ* ≠ 1 and *λ* ≠ 16. There are also another six strange fixed points (a fixed point is called strange if it does not correspond to any root of the polynomial), whose analytical expression, depending on *λ*, is very complicated.

As we will see in the following, not only the number but also the stability of the fixed points depend on the parameter of the family. The expression of the differential operator, necessary for analyzing the stability of the fixed points and for obtaining the critical points, is

(6) Op′(z,λ)=−4z3(1+z)4(−(1+z)4+λ(1−z+z2−z3+z4))(1+4z+6z2+4z3−(−1+λ)z4)2.

As they come from the roots of the polynomial, it is clear that the origin and *∞* are always superattractive fixed points, but the stability of the other fixed points can change depending on the values of the parameter *λ*. In the following result we establish the stability of the strange fixed point *z* = 1.

**Theorem 2**

The character of the strange fixed point *z* = 1 is as follows.

- If |*λ* − 16| > 64, then *z* = 1 is an attractor and it cannot be a superattractor.
- When |*λ* − 16| = 64, *z* = 1 is a parabolic point.
- If |*λ* − 16| < 64, being *λ* ≠ 1 and *λ* ≠ 16, then *z* = 1 is a repulsor.

**Proof**

It is easy to prove that

(7) $$\begin{array}{c} O_{p}^{\prime }\left(1,\lambda \right)=\frac{64}{16-\lambda }. \end{array}$$

So,

(8) $$\begin{array}{c} \left|\frac{64}{16-\lambda }\right|\leq 1\;\text{is}\;\text{equivalent}\;\text{to}\;64\leq \left|16-\lambda \right|. \end{array}$$

Let us consider *λ* = *a* + *ib* an arbitrary complex number. Then,

(9) $$\begin{array}{c} 64^{2}\leq 16^{2}-32a+a^{2}+b^{2}. \end{array}$$

That is,

(10) $$\begin{array}{c} {\left(a-16\right)}^{2}+b^{2}\geq 64^{2}. \end{array}$$

Therefore,

(11) $$\begin{array}{c} \left|O_{p}^{\prime }\left(1,\lambda \right)\right|\leq 1\;\text{iff}\;\left|\lambda -16\right|\geq 64. \end{array}$$

Finally, if *λ* verifies |*λ* − 16| ≤ 64, then |*O*_*p*_′(1, *λ*)| > 1 and *z* = 1 is a repulsive point, except if *λ* = 1 or *λ* = 16, values for which *z* = 1 is not a fixed point.

The critical points are *z* = 0, *z* = *∞*, and *z* = −1 (for *λ* ≠ 0), and

(12) cr1(λ)=14[1+1λ−1−β−2−5λ(6−7λ+λ2)(λ−1)3−(4+λ)βλ−1],cr2(λ)=14[1+1λ−1−β+2−5λ(6−7λ+λ2)(λ−1)3−(4+λ)βλ−1],cr3(λ)=14[1+1λ−1+β−2−5λ(6−7λ+λ2)(λ−1)3+(4+λ)βλ−1],cr4(λ)=14[1+1λ−1−β+2−5λ(6−7λ+λ2)(λ−1)3+(4+λ)βλ−1],

where *λ* ≠ 1 and $\beta =\left(\sqrt{5}\sqrt{{\left(\lambda -1\right)}^{2}}\sqrt{\lambda \left(4+\lambda \right)}\right)/{\left(\lambda -1\right)}^{2}$.

The relevance of the knowledge of the free critical points (critical points different from the associated with the roots) is the following known fact: each invariant Fatou component is associated with, at least, one critical point.

**Lemma 3**

Analyzing the equation *O*_*p*_′(*z*, *λ*) = 0, one obtains the following.

- If *λ* = 0, there is no free critical points of operator *O*_*p*_(*z*, 0).
- If *λ* = 16, then there are four free critical points: *z* = −1, $cr_{1}\left(16\right)=(1/4)\left(-(4/3)-(8\sqrt{2}/3)i\right)$, $\;cr_{2}\left(16\right)=(1/4)\left(-(4/3)+(8\sqrt{2}/3)i\right)$, and *cr*_3_(16) = *cr*_4_(16) = 1.
- If *λ* = −4, then there are three different critical points: *z* = −1, *cr*_1_(−4) = *cr*_3_(−4) = −*i*, and *cr*_2_(−4) = *cr*_4_(−4) = *i*.
- In case of *λ* = 1, the set of critical points is $\left\{-1,-(1/2)+(\sqrt{3}/2)i,-(1/2)-(\sqrt{3}/2)i\right\}$.
- In any other case, *z* = −1, *cr*_1_(*λ*), *cr*_2_(*λ*), *cr*_3_(*λ*), and *cr*_4_(*λ*) are the free critical points.

Moreover, it can be proved that all free critical points are not independent, as *cr*_1_(*λ*) = 1/*cr*_2_(*λ*) and *cr*_3_(*λ*) = 1/*cr*_4_(*λ*).

Some of these properties determine the complexity of the operator, as we can see in the following results.

**Theorem**

The only member of the family whose operator is always conjugated to the rational map *z* ^4^ is the element corresponding to *λ* = 0.

**Proof**

From (5), we denote *n*(*z*) = 1 − *λ* + 4*z* + 6*z* ^2^ + 4*z* ^3^ + *z* ^4^ and *d*(*z*) = 1 + 4*z* + 6*z* ^2^ + 4*z* ^3^ − (−1 + *λ*)*z* ^4^. By factorizing both polynomials, we can observe that the unique value of *λ* verifying *n*(*z*) = *d*(*z*) is *λ* = 0.

In fact, the element of Kim's class corresponding to *λ* = 0 is Ostrowski's method. So, it is the most stable scheme of the family, as there are no free critical points, and the iterations can only converge to any of the images of the roots of the polynomial. This is the same behavior observed when Ostrowski's scheme was analyzed by the authors as a member of King's family in [14].

**Theorem**

The element of the family corresponding to *λ* = 1 is a fifth-order method whose operator is the rational map

(13) $$\begin{array}{c} O_{p}\left(z,\lambda \right)=\frac{z^{5}\left(2+z\right)\left(2+2z+z^{2}\right)}{\left(1+2z\right)\left(1+2z+2z^{2}\right)}. \end{array}$$

**Proof**

From directly substituting *λ* = 1 in the rational operator (5), (13) is obtained, showing that *z* = 1 is not a fixed point in this particular case. Moreover,

(14) $$\begin{array}{c} O_{p}^{\prime }\left(z,\lambda \right)=\frac{20z^{4}{\left(1+z\right)}^{4}\left(1+z+z^{2}\right)}{{\left(1+2z\right)}^{2}{\left(1+2z+2z^{2}\right)}^{2}}, \end{array}$$

and there exist only three free critical points.

Then, in the particular case *λ* = 1, the order of convergence is enhanced to five, and although there are three free critical points, they are in the basin of attraction of zero and infinity, as the strange fixed points are all repulsive in this case. So, it is a very stable element of the family with increased convergence in case of quadratic polynomials.

### 2.1. Using the Parameter and Dynamical Planes

From the previous analysis, it is clear that the dynamical behavior of the rational operator associated with each value of the parameter can be very different. Several parameter spaces associated with free critical points of this family are obtained. The process to obtain these parameter planes is the following: we associate each point of the parameter plane with a complex value of *λ*, that is, with an element of family (2). Every value of *λ* belonging to the same connected component of the parameter space gives rise to subsets of schemes of family (2) with similar dynamical behavior. So, it is interesting to find regions of the parameter plane as much stable as possible, because these values of *λ* will give us the best members of the family in terms of numerical stability.

**Figure 1:**
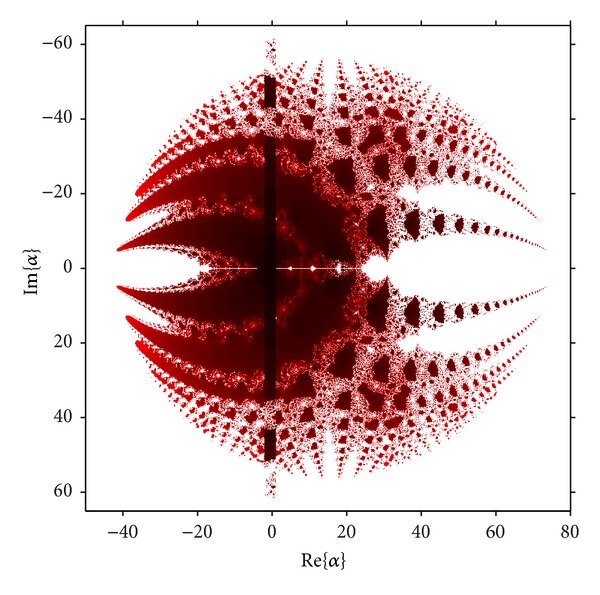
Parameter plane *P*_1_ associated with *z* = cr_*i*_, *i* = 1,2.

As cr_1_(*λ*) = 1/cr_2_(*λ*) and cr_3_(*λ*) = 1/cr_4_(*λ*) (see Lemma 3), we have at most three independent free critical points. Nevertheless, *z* = −1 is preimage of the fixed point *z* = 1 and the parameter plane associated with this critical point is not significative. So, we can obtain two different parameter planes, with complementary information. When we consider the free critical point cr_1_(*λ*) (or cr_2_(*λ*)) as a starting point of the iterative scheme of the family associated with each complex value of *λ*, we paint this point of the complex plane in red if the method converges to any of the roots (zero and infinity) and they are white in other cases. Then, the parameter plane *P*_1_ is obtained; it is shown in Figure 1. 

**Figure 2:**
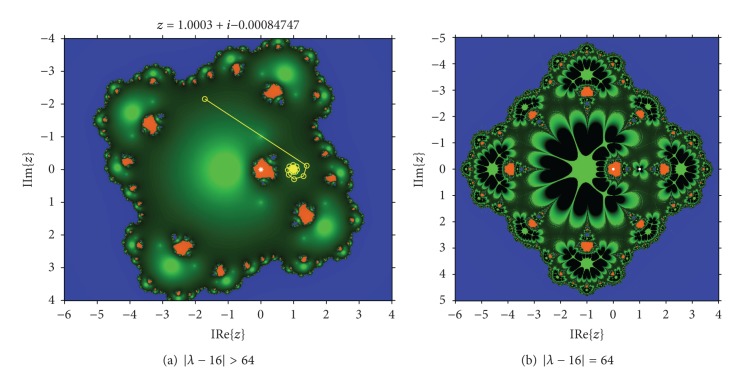
Dynamical planes for *λ* verifying |*λ* − 16| ≥ 64.

This figure has been generated for values of *λ* in [−50,80]×[−65,65], with a mesh of 2000 × 2000 points and 400 iterations per point. In *P*_1_ the disk of repulsive behavior of *z* = 1 is observed, showing different white regions where the convergence to *z* ≠ 0 and *z* ≠ *∞* has been reached. An example of a dynamical plane associated with a value of the parameter is shown in Figure 2(a), where three different basins of attraction appear, two of them of the superattractors 0 and *∞* and the other of *z* = 1, that is, a fixed attractive point. It can be observed how the orbit (in yellow in the figure) converges asymptotically to the fixed point. Also in Figure 2(b), the behavior in the boundary of the disk of stability of *z* = 1 is presented, where this fixed point is parabolic. An orbit would tend to the parabolic point alternating two “sides” (up and down of the parabolic point in this case). 

The generation of dynamical planes is very similar to the one of parameter spaces. In case of dynamical planes, the value of parameter *λ* is constant (so the dynamical plane is associated with a concrete element of the family of iterative methods). Each point of the complex plane is considered as a starting point of the iterative scheme, and it is painted in different colors depending on the point which it has converged to. A detailed explanation of the generation of these graphics, joint with the Matlab codes used to generate them, is provided in Section 3.

**Figure 3:**
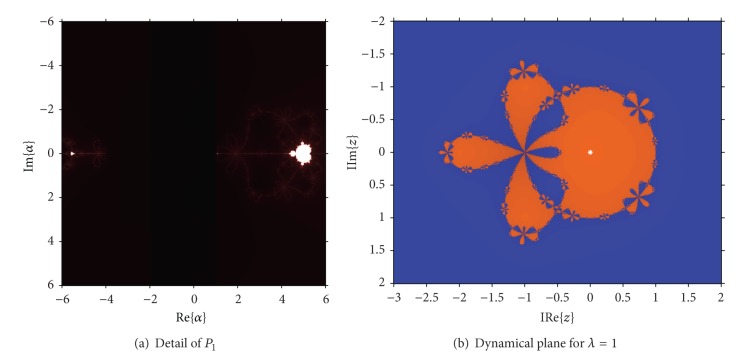
Around the origin.

In Figure 3(a), a detail of the region around *λ* = 0 of *P*_1_ can be seen. Let us notice that region around the origin is specially stable, specifically the vertical band between −4 and 1 (see also Figure 3(b)). 

**Figure 4:**
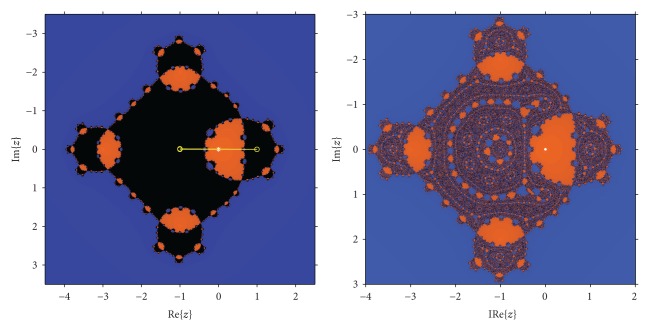
Around *λ* = 16.

In fact, for *λ* = 0, the associated dynamical plane is the same as the one of Newton's, that is, it is composed by a disk and its complementary in *ℂ*. Around the origin is also very stable, with two connected components in the Fatou set. When *λ* = 16, *z* = 1 is not a fixed point (see Theorem 2) and {−1,1} define a periodic orbit of period 2 (see Figure 4(a)). The singularity of this value of the parameter can be also observed in Figure 4(b), in which a dynamical plane for *λ* = 15.9 − 0.2*i* is presented, showing a very stable behavior with only two basin of attraction, corresponding to the image of the roots of the polynomial by the Möbius map. 

It is also interesting to note in Figure 3(a) that white figures with a certain similarity with the known Mandelbrot set appear. Their antennas end in the values *λ* = −4 and *λ* = 1, whose dynamical behavior is very different from the near values of the parameter, as it was shown in Lemma 3.

**Figure 5:**
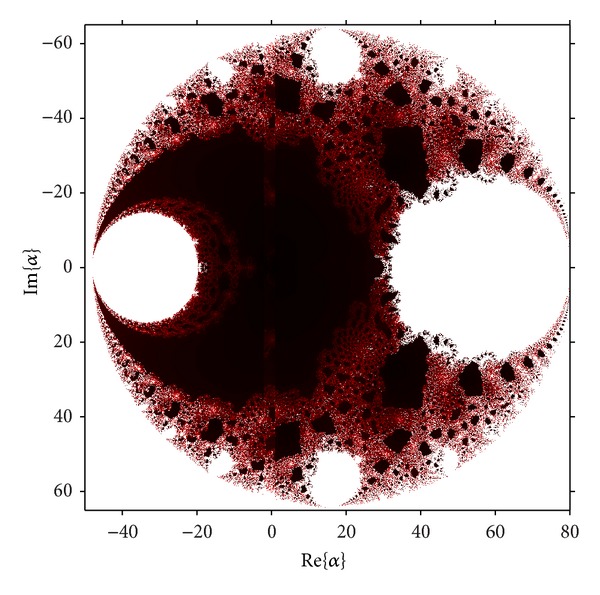
Parameter space *P*_2_ associated with *z* = cr_*i*_, *i* = 3,4.

A similar procedure can be carried out with the free critical points, *z* = cr_*i*_, *i* = 3,4, obtaining the parameter planes *P*_2_, shown in Figure 5. 

**Figure 6:**
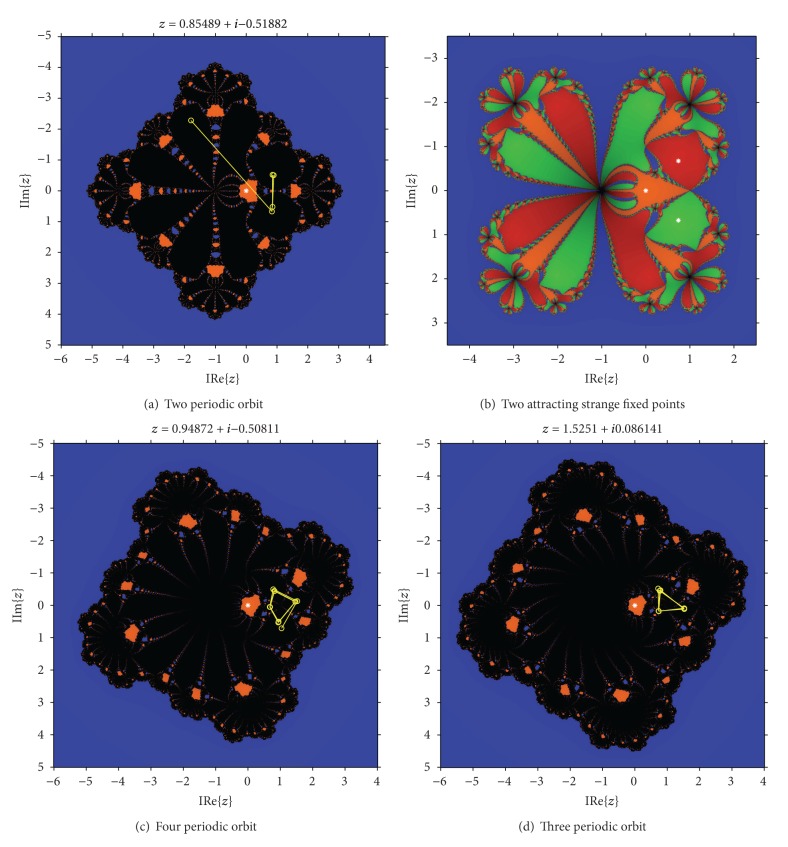
Some dynamical planes from *P*_2_.

As in case of *P*_1_, the disk of repulsive behavior of *z* = 1 is clear, and inside it different “bulbs” appear, similar to disks. The biggest on the left of the real axis corresponds to the set of values of *λ* where the fixed point *z* = 1 has bifurcated in a periodic orbit of period two, as can be seen in Figure 6(a). In the right of the real axis a bulb is the loci of two conjugated strange fixed points; see Figure 6(b).

The bulbs on the top (see Figure 6(c)) and on the bottom of the imaginary axis correspond to periodic orbits of period 4. The rest of the bulbs surrounding the boundary of the stability disk of *z* = 1 correspond to regions where periodic orbits of different periods appear. In fact, we can observe in Figure 6(d)) a periodic orbit of period 3, obtained from *λ* = 50 + 50*i*. By applying Sharkovsky's theorem (see [15]), we can affirm that periodic orbits of arbitrary periodicity can be found.

**Pseudocode 1**

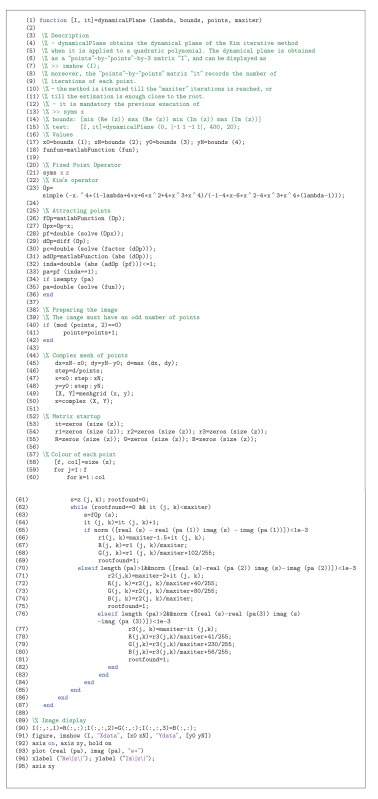

**Pseudocode 2**

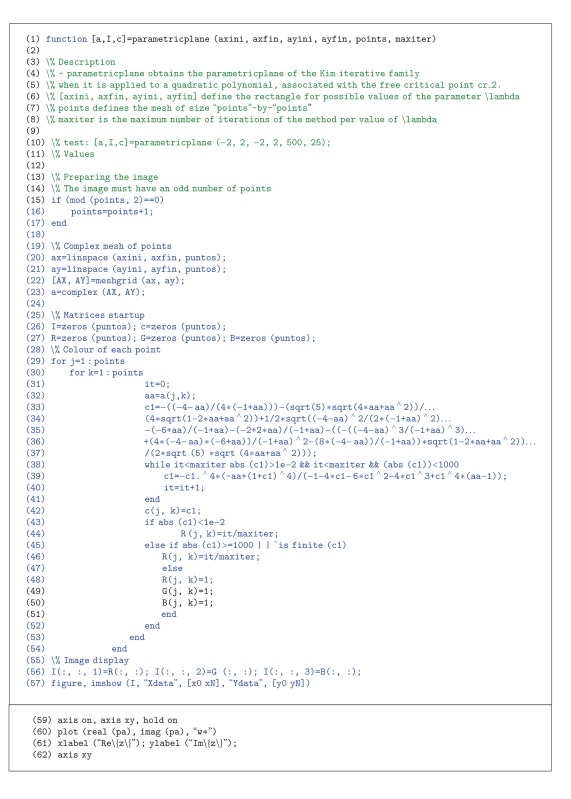

( 5 9 ) axis on, axis xy , hold on
                                
( 60 ) plot (real (pa), imag (pa), “w ∗”)
( 6 1 ) x label (“Re *∖*{z *∖*}”); y label (“Im *∖*{z *∖*}”);
( 6 2 ) axis xy

## 3. MATLAB Planes Code

The main goal of drawing the dynamical and parameters planes is the comprehension of the family or method behavior at a glance. The procedure to generate a dynamical or a parameters plane is very similar. However, there are small differences, so both cases are developed below.

### 3.1. Dynamical Planes

From a fixed point operator, that associates a polynomial with an iterative method, the dynamical plane illustrates the basins of attraction of the operator. The orbit of every point in the dynamical plane tends to a root (or to the infinity); this information and the speed that the points tend to the root can be displayed in the dynamical plane. In our pictures, each basin of attraction is drawn with a different color. Moreover, the brightness of the color points the number of iterations needed to reach the root of the polynomial.

Pseudocode 1 covers the Kim's fixed point operator, when it is applied to a quadratic polynomial. This code has been utilized to generate the dynamical planes of several papers, as [9, 10, 14] or [17].

The code is divided into five different parts.

1. Values (lines 17-18): the bounds are renamed and the symbolic function introduced as fun is translated into an anonymous function, recallable by the output handle.
2. Fixed point operators (line 23).
3. Calculation of attractive fixed points (lines 26–36).
4. Image creation (lines 39–94): once the fixed point operator and the attracting points are set, the next step consists of the determination of the basins of attraction. The combination of the input parameters bounds and points set the resolution of the image, and it establishes the mesh of complex points (lines 39–50).
5. Lines 58–87 are devoted to assign a color to each starting point. It depends on the basin of attraction and the number of iterations needed to reach the root. If the orbit tends to the attracting point set in the first index of line 35, the point is pictured in orange, as lines 67–69 show; for the second and third cases, the point is pictured in blue (lines 72–74) and green (lines 78–80), respectively. Otherwise, the point is not modified, so its color is black.
6. As the number of iterations needed to reach convergence increases, its corresponding color gets closer to white (black in the decreasing case). A coefficient in each case (lines 66, 71, and 77) is high if the number of iterations is low, and the RGB values are greater than in the slow orbit instance.
7. Image display (lines 90–94): the image display is based on the imshow command. Images are usually displayed in matrix form (from top to bottom and from left to right). In this case, the image is composed of complex points, so the natural display is the Cartesian one (from bottom to top and from left to right). With this purpose, axis xy is written in line 95.

Once the program is executed, the output values are the image I and the number of iterations of each point it. Our recommendation is the use of the surf command to plot the number of iterations, in combination with the shading one.

In order to apply the introduced code to different fixed point operators, the only part to be changed is the fixed point operators corresponding one. If the method can converge to more than three points, just add another else if structure (as lines 79–84) and set a color as many times as necessary.

### 3.2. Parameter Planes

Pseudocode 2 is divided into five different parts.

1. Generation of the mesh of values of *λ* (lines 19–23).
2. Matrices startup (line 26-27).
3. Iterative process (lines 31–44). The value of the critical point depends on *λ*, so in lines 33–37 is obtained. Its orbit is calculated in lines 38–41.
4. Colors assignment (lines 43–51). If the critical point converges, it is drawn by a red-family color (lines 43–46)—otherwise, it is plotted in white (lines 47–51).
5. Image display (lines 55–61): the image display is based on the imshow command. Images are usually displayed in matrix form (from top to bottom and from left to right). In this case, the image is composed of complex points, so the natural display is the Cartesian one (from bottom to top and from left to right). With this purpose, axis xy is written in line 62.

Once the program is executed, the output values are the image I and the number of iterations of each point it. Our recommendation is the use of the surf command to plot the number of iterations, in combination with the shading one.

In order to apply the introduced code to different fixed point operators, the only part to be changed is the fixed point operators corresponding one. If the method can converge to more than three points, just add another else if structure (as lines 79–84) and set a color as many times as necessary.

## 4. Conclusions

We have analyzed the dynamical properties of the parametric Kim's family showing stability regions and elements of the family with interesting dynamical behavior but bad numerical features. The main tools used to get this aim are the parameter and dynamical planes implemented in Matlab, whose code is presented in the last section.
